# A new midline closure technique without skin sutures: Long-term outcomes of primary repair of pilonidal sinus disease

**DOI:** 10.23938/ASSN.1073

**Published:** 2024-04-17

**Authors:** Hüseyin Taş, Furkan Karahan

**Affiliations:** 1 Department of General Surgery Izmir Katip Celebi University Atatürk Education and Research Hospital Izmir Turkey; 2 Erciş Şehit Rıdvan Çevik State Hospital Department of General Surgery Van Turkey

**Keywords:** Sinus Pilonidal, Wound Closure Techniques, Sutureless Surgical Procedures, Treatment Outcome, Seno Pilonidal, Técnicas de Cierre de Heridas, Procedimientos Quirúrgicos sin Sutura, Resultado del Tratamiento

## Abstract

**Background:**

Currently, the focus regarding pilonidal sinus disease is put on the treatment techniques. The aim of the study is to compare postoperative long-term complications and recurrence of two surgical techniques.

**Material and methods:**

From February 2015 to December 2020, male patients with pilonidal sinus disease attended at two general surgery outpatient centers were randomly assigned to either Group 1 (n=80; excision and primary closure) or Group 2 (n=80; excision and midline closure without skin sutures). Patients with recurrent or complicated pilonidal sinus or with prior surgical procedures were excluded from the study. Intergroup postoperative results and recurrence throughout the follow-up period were analyzed.

**Results:**

Significant decrease (p<0.001) in the duration of the surgical procedure (35 to 25 minutes), length of hospital stay (one day to the day of the surgery), and of the time required to return to work (15 to 12 days) was seen for Group 2 patients. The complication rate (wound infection and seroma) was lower in Group 2 compared to Group 1 (n = 3; 3.7% vs n = 10; 12.5%; p = 0.014). During the five-year mean follow-up, five patients (6.2%) in Group 1 had recurrence compared to none in Group 2 (p = 0.023).

**Conclusions:**

Midline primary closure method without skin sutures - easy to learn and implement and has no complication or recurrence in the long-term follow-up - may be an ideal method in cases where excision and primary repair is planned, especially in patients with sinus orifices located in the midline.

## RESUMEN

**Fundamento:**

Hoy en día, el foco sobre la enfermedad del seno pilonidal se centra en las técnicas de tratamiento. El objetivo del estudio es comparar dos técnicas quirúrgicas diferentes respecto a las complicaciones posoperatorias y la recidiva a largo plazo.

**Metodología:**

Estudio longitudinal de pacientes masculinos que acudieron a la clínica ambulatoria de cirugía general desde febrero de 2015 hasta diciembre de 2020 por enfermedad del seno pilonidal. Los pacientes se aleatorizaron en Grupo 1 (n=80, escisión más cierre primario) y Grupo 2 (n=80, escisión más cierre de la línea media sin suturas cutáneas) y se compararon los resultados. postoperatorios y las recidivas durante el seguimiento. Se excluyeron los pacientes con *sinus* pilonidal recurrente o complicado o con cirugía previa.

**Resultados:**

Los pacientes del Grupo 2 mostraron un tiempo significativamente menor (p<0,001) de cirugía (25 minutos vs 35), de estancia hospitalaria (0 vs 1 día) y de baja médica (12 días vs 15). La frecuencia de complicaciones (infección y seroma) fue menor que en el Grupo 1 (3,7% vs 12,5%; p = 0,014). Durante los cinco años de seguimiento medio, cinco pacientes del Grupo 1 recidivaron (6,2%) frente a ninguno del Grupo 2 (p=0,023).

**Conclusiones:**

El cierre primario de la línea media sin suturas cutáneas, fácil de aprender e implementar y que no presenta complicaciones ni recidivas en el seguimiento a largo plazo, puede ser un método ideal en los casos en los que se planifica escisión más reparación primaria, especialmente en aquellos con orificios sinusales ubicados en la línea media.

## INTRODUCTION

Pilonidal sinus is a condition most commonly found in the hair follicles of the natal cleft of the sacrococcygeal area. It was first described by Mayo^1^ and Hodge introduced the name *pilonidal* (*pilus* meaning hair and *nidus* meaning core in Latin)^2^ Patey and Scarff suggested that the condition might be caused by a granulomatous reaction due to the penetration of hair into the subcutaneous tissue^3^.

A number of theories on what may cause pilonidal sinus have been proposed; however, the focus is currently put on the techniques used for its treatment. The ideal surgery should be simple with a short hospital stay and low recurrence rate. There should be minimal pain and wound care with rapid return to normal activity. There are significant differences between the surgical techniques with regard to technique simplicity and morbidity, hospitalization, return to work, cost, and especially recurrence. Total excision and the primary repair method is the most common technique; the disadvantages of this method includes longer hospital stays (due to high rates of seroma development and infection) and higher rates of recurrence. Some suggested recurrence-related factors are hemorrhagic collection, seroma, and pouch formation. The thick suture materials used in primary closure cannot be removed for at least two weeks due to mobility and tension in the affected site. The orifices caused by skin reaction and sutures are thought to be associated to recurrence^4^^,^^5^.

A new closure technique has been developed aiming to avoid potential dead spaces and orifices that may result from suture and repair. The aim of this prospective study was to compare a series of variables of interest (e.g., recurrence) between two methods: excision and primary closure and a new technique of excision and midline closure free from skin sutures.

## PATIENTS AND METHODS

Prospective longitudinal study that included male patients who attended for pilonidal sinus disease to the General Surgery outpatient clinic of İzmir Katip Çelebi University Atatürk Training and Research Hospital (Turkey) and Gulhane Military Medical Academy Hospital General Surgery Clinic (Turkey) from February 2015 to December 2020.

### Patients

Patients with sinus orifices located in the midline were included, whereas patients with recurrent or complicated pilonidal sinus or with prior surgery were excluded. Study participants with acute abscess formation were first treated with drainage and antibiotic therapy; next and at least 20 days later the surgical procedure was performed.

Patients were randomly assigned to either one of the two surgical techniques: those in Group 1 underwent excision and primary closure (standard method) and those in Group 2 underwent excision and midline closure without skin sutures. The results between both methods were compared.

### Surgical technique

All the procedures were performed under local anesthesia. Patients were given antibiotic prophylaxis: a single dose of cefazolin (1 g intravenous injection) prior to surgery. Patients were made to lie in prone position with the buttocks held apart by strapping.

**Figure 1:**
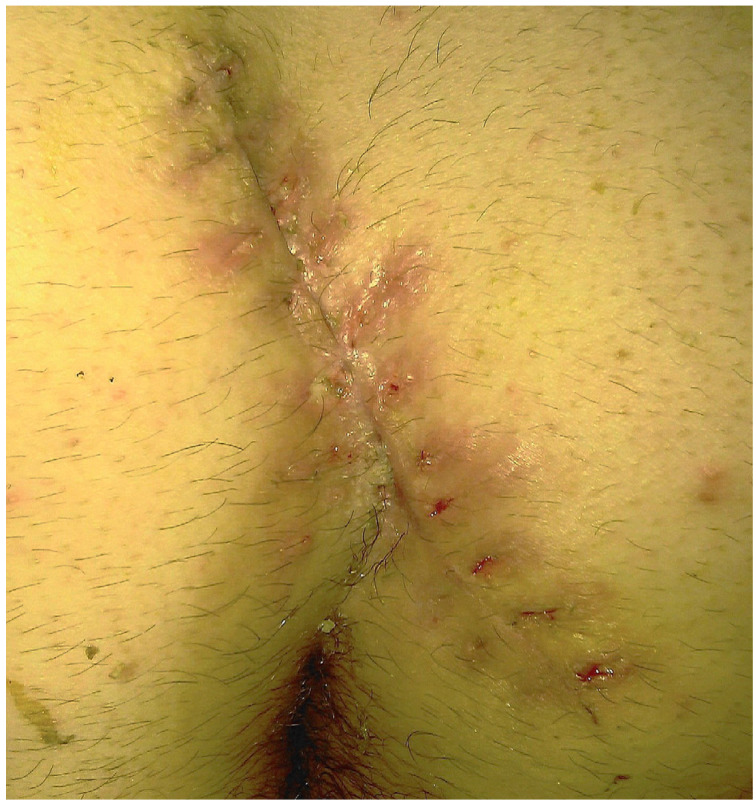
Image of the orifices caused by skin reaction and sutures in a patient from Group 1 two weeks after surgery.

The surgical procedure used for Group 1 participants was an elliptical excision of all diseased tissue down to the postsacral fascia. The subcutaneous tissue was advanced with 2/0 polyglactic acid and the skin was sutured with 2/0 polypropylene. Primary closure was performed on two layers. Sutures were removed after two weeks (Figure 1).

**Figure 2:**
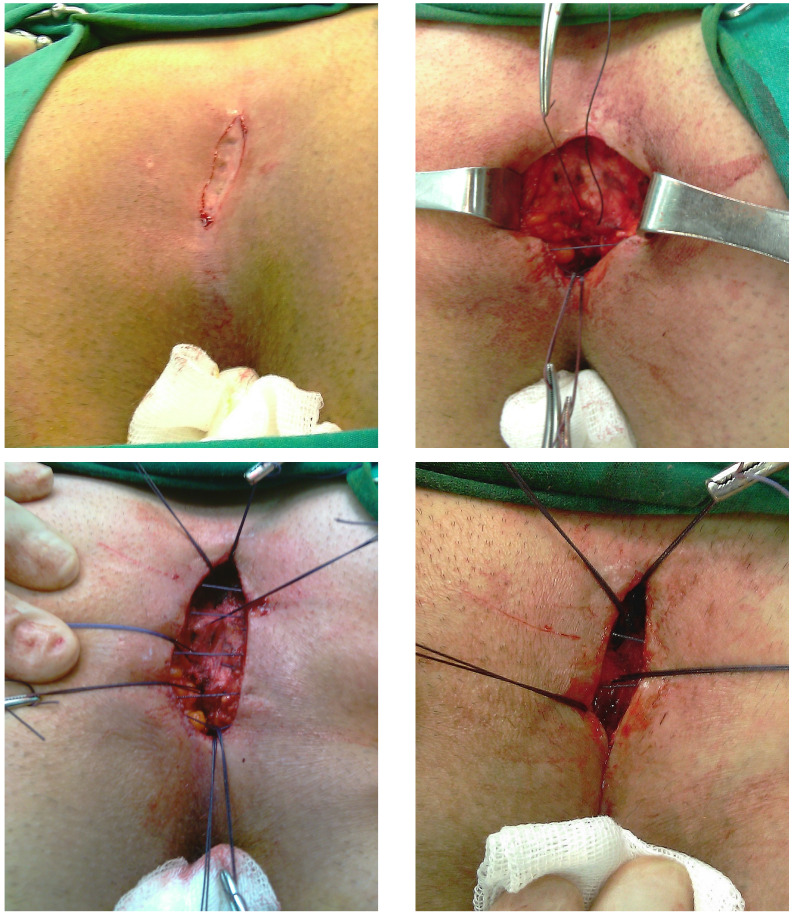
Steps of the new suturing technique: **A.** Elliptic incision. **B.** Suturing method applied through the respective layers including postsacral fascia, subcutaneous layer, opposite subcutaneous tissue, and postsacral fascia. **C.** An image showing the completed sutures. **D.** Knot placed at the mid-line of the fascia.

The surgical procedure used for Group 2 patients was again an elliptical excision of all diseased tissue without reaching the postsacral fascia with minimal tissue loss. The incision was slightly oblique to help mobilization of wound sides and reduce the tension at the closure of subcutaneous adipose tissue; 2/0 polyglactic acid suture was used. Starting from the mid postsacral fascia, the sutures were placed between 1 and 1.5 cm apart going up subcutaneously in the first wound side and then down subcutaneously in the opposite side, up mid postsacral fascia again and closing one layer so that the knot was placed at the midline of the fascia (Figure 2). No drainage was used in any of the patients. Compressing dressings with multiple gauzes were used for hemostasis and to avoid any dead spaces.

### Postoperative assessment and follow-up

Patient follow-up was carried out in the supine position. Discharge criteria were stable vital values without bleeding or postoperative pain (visual analog scale score ≤3).

After wound dressings were changed three times a day on alternate days, patients were advised to avoid pressure on the operated site for 15 days and clean the wound area with warm-hot water and epilator creams for six months. The wound site was assessed on Day 1 and Week 1 post-surgery. Patients were examined for early recurrences one, three, and six months after surgery, and further follow-ups were done over the phone.

### Variables

The following variables were recorded and inter-group statistical comparisons performed:

- Demographic: age (years), sex (all patients were male);
- Clinical: symptoms (secretion, pain, none), duration of the symptoms (months), abscess development (yes, no);
- Surgical: duration of surgery (minutes), length of hospital stay (days), complications as wound dehiscence, wound infection (inflammation of the surgical field with microorganisms involved), seroma (fluid accumulation in the dead space in the surgical area and/or hematoma formation under the flap suture), and/or hematoma;
- Progress: healing (closure of the wound without any complications within the first 30 days after the surgery, time to return to work (days), recurrence of pilonidal sinus disease (31 days or later opening of a healed wound, as well as any openings in the scar, nearby skin, or formation of a painful subcutaneous mass in the intergluteal area).

### Ethical statement

The Ethics Committee approval for this study was obtained from the Gulhane Military Medical Academy Hospital Ethics Committee (GATA-GOKAEK 2012/2009). All participants/legal representatives signed the written informed consent. This study was performed in accordance with the ethical standards laid down in the 1964 Declaration of Helsinki.

### Statistical analysis

Quantitative data are shown as mean (standard deviation) and compared using independent Student’s t-test; qualitative variables are shown as frequency and percentage and compared using the Chi-square test. An *a priori* power analysis was performed as a component of design to estimate the required total sample size as a function of power 1- β = 0.80, with a medium effect size of 0.55 and α = 0.05. Power calculation was computed using G power version 2 (Franz Faul and Edgar Erdfelder). The data were analyzed using the SPSS 24.0 statistical package. All statistical analyses were two-tailed and a p-value <0.05 was considered statistically significant.

## RESULTS

One hundred and sixty male patients enrolled in the study; mean age was 25 years (range: 18-34). The main symptom was secretion, followed by pain, with a mean duration of symptoms of two years. Baseline characteristics of both groups were similar (Table 1).

**Table 1:** Demographic characteristics and preoperative symptoms

Variables	Group 1	Group 2	p (χ^2^)
Age (years), *mean (SD)*	26 (8.4)	24 (7.6)	0.245*
Sex (male), *n (%)*	80 (100)	80 (100)	
Duration of symptoms (months), *mean (SD)*	24 (±4.64)	23 (±4.97)	0.152*
*Symptoms, n (%)*			0.853
Secretion	60 (75.00)	58 (72.50)	
Pain	50 (62.50)	45 (56.25)	
None	9 (11.25)	12 (15.00)	
Abscess development, *n (%)*	46 (57.50)	40 (50.00)	0.341

Group 1: excision and primary closure; Group 2: excision and midline closure without skin sutures; SD: standard deviation; *: Student’s t-test.

Group 2 patients showed significantly shorter mean duration of surgical procedure (10 minutes), hospital stay (one day), and time required to return to work (three days) (Table 2).

**Figure 3:**
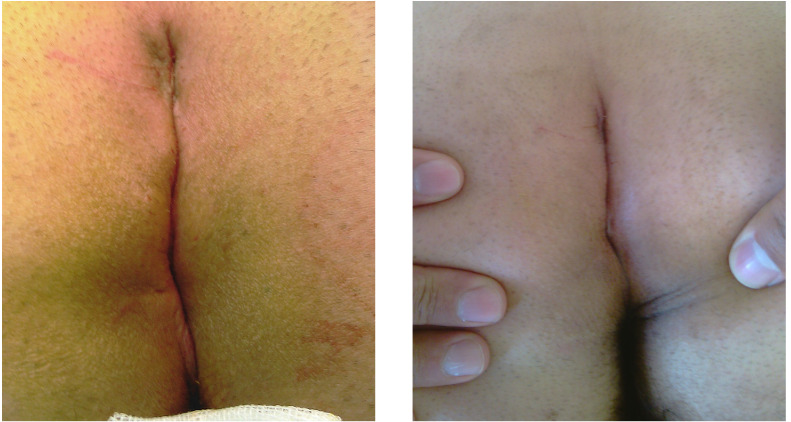
Images of the wound area in Day 1 (**A**) and Week 1 after surgery (**B**) from a Group 2 patient.

The wound site was assessed on Day 1 and Week1 postoperatively (Figure 3). Wound closure and early recurrence follow-up were performed one, three, and six months after the surgery. Subsequent follow-ups (long-term complications) were carried out over the phone.

The complication rate in Group 1 was significantly higher compared to that in Group 2 (n = 10; 2.5% vs n = 3; 3.7%; p = 0.014). In Group 1, six patients developed wound infection, from which five improved with antibiotic treatment; in the other patient, skin sutures were released for additional drainage and the wound was left for secondary healing. Puncture was adequate in one out of two patients with seroma and, in the other, wound was left for secondary healing after removing approximately one third of the skin sutures. Thus, the sutures of two patients were inadequate. No seroma was detected in participants from Group 2 and three developed wound infection; the former responded to antibiotic treatment without requiring any additional surgical procedure.

After a mean follow-up period of 60 months (SD: 7.73; range = 36 - 84), five patients from Group 1 (6.2%) experienced recurrence and none in Group 2 (Table 2).

**Table 2:** Outcomes and complications

Variables	Group 1	Group 2	p (χ^2^)
Duration of surgery (minutes), *mean (SD)*	35 (±2.85)	25 (±2.85)	<0.001*
Length of hospital stay (days), *mean (SD)*	1(±2.75)	0 (±0)	<0.001*
Time of return to work (days)¸ *mean (SD)*	15 (±3.47)	12 (±0.69)	<0.001*
Complications in the early postoperative period, *n (%)*	10 (12.5)	3 (3.7)	0.014
Infection at the wound area, *n (%)*	6	3	
Suture dehiscence, *n (%)*	2	0	
Seroma, *n (%)*	2	0	
Recurrence, *n (%)*	5 (6.2)	0	0.023

Group 1: excision and primary closure; Group 2: excision and midline closure without skin sutures; SD: standard deviation; *: Student’s t-test.

**Figure 4:**
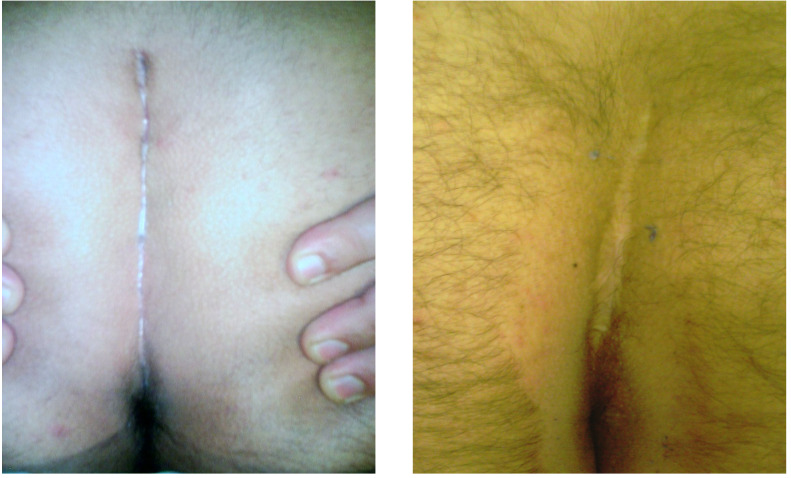
Postoperative images of the wound area in two patients from Group 2. **A**. Three months after surgery. **B**. Five years after surgery.

Figure 4 shows the appearance of the area five years after the surgical procedure.

## DISCUSSION

Pilonidal sinus occurs mainly in young adults, with reported incidence rates showing variations in different countries^6^. The pathogenesis of these lesions is uncertain, but the most likely causative factor is implantation of loose hairs into a pre-existing perianal cutaneous defect or scar. An accumulation of hair in the natal cleft, insertion of hair through a macerated skin part causing foreign body reaction, and development of infection are thought to be responsible^7^. However, one of the reasons for recurrence in previously operated patients with pilonidal sinus is the iatrogenic orifices due to skin sutures^3^.

Numerous techniques have been described for the treatment of pilonidal disease. Closure at the midline is the preferred method with acceptable rates of recurrence in uncomplicated cases and rapid healing^8^. In this technique, the wound sides are advanced towards the postsacral fascia without requiring separate skin sutures and no pouch. Therefore, midline repair without the use of skin sutures helps protect the anatomic structures with maximum cosmetic benefit.

Development of seroma has been reported in 2.2% to 8% of the cases who underwent total excision and primary repair^9^; seroma is caused because of the dead space left by the excision and the inability to close it during the repair, as well as by inadequate hemostasis. In our study, seroma develops in two patients from Group 1 and none from Group 2 (significant intergroup difference). In this technique, after a minimal excision and ensuring that each suture passes all the way through the subcutaneous tissue, complete closure of the dead space and hemostasis is achieved. Consequently, development of seroma is reduced or prevented. Furthermore, the non-use of skin sutures prevents tension on the incision line and allows free drainage of the seroma that could develop during the early postoperative period.

Wound infection rates range from 7.3% to 38%^10^^-^^12^. In our study, Group 2 participants display a significantly lower wound infection rate compared to those from Group 1. It is difficult to show the effect on wound infection rates of the new suture technique; however, early postoperative skin problems, including infection and excoriation, are partly prevented as there are no inflamed suture orifices due to the non-use of skin sutures. Moreover, no skin suture-related tissue circulation problems occur around the wounds.

Mean length of hospital stay of patients who undergo excision and primary closure under local anesthesia has been reported to be 2.6 days^12^^-^^14^. In our study, mean length of hospital stay is 0 days for Group 2 and 1 day for Group 1. A possible explanation for this difference may be the sutures placed on the skin, which prolong the duration of dermal pain in Group 1 patients. The shorter duration of hospital stay in our study compared to published series may be due to our strategy of discharging as quickly as possible patients who receive local anesthesia.

Reported recurrence rates range from 0% to 37.5%^15^^-^^17^. In our study, recurrence is seen in five patients from Group 1 and none in those from Group 2. The significant intergroup difference in recurrence may be due to minimal excision, avoiding seroma because of the closure technique, absence of suture orifices on the skin, and the low rate of wound infection due to personal hygiene and epilators. Another advantage of the new method is the possibility of a second operation due to minimal loss of tissue and maximum preservation of the anatomy in case of recurrence.

A limitation of this study is that only male patients were enrolled. One of the two participating centers is a hospital where male soldiers do compulsory military service.

In conclusion, primary midline closure without skin sutures, a simple and easy method with a low recurrence rate, may be an ideal method in cases with sinus orifices located at the midline and for which excision and primary repair are planned. The method is free from long-term complications and/or recurrence. However, further studies are required to assess the availability of this method in patients with complicated and recurrent pilonidal sinus disease.
